# miR-582-5p Is Upregulated in Patients with Active Tuberculosis and Inhibits Apoptosis of Monocytes by Targeting FOXO1

**DOI:** 10.1371/journal.pone.0078381

**Published:** 2013-10-24

**Authors:** Yanhua Liu, Jing Jiang, Xinjing Wang, Fei Zhai, Xiaoxing Cheng

**Affiliations:** Division of Research, Laboratory of Tuberculosis Prevention and Treatment of PLA, Institute of Tuberculosis, 309 Hospital, Beijing, China; University of San Francisco, United States of America

## Abstract

Macrophage apoptosis is a host innate defense mechanism against tuberculosis (TB). In this study, we found that percentage of apoptotic cells in peripheral blood monocytes from patients with active TB was lower than that from healthy controls (p<0.001). To understand whether microRNAs can modulate apoptosis of monocytes, we investigated differentially expressed microRNAs in patients with active TB. miR-582-5p was mainly expressed in monocytes and was upregulated in patients with active TB. The apoptotic percentage of THP-1 cells transfected with miR-582-5p mimics was significantly lower than those transfected with negative control of microRNA mimics (p<0.001), suggesting that miR-582-5p could inhibit apoptosis of monocytes. To our knowledge, the role of miR-582-5p in regulating apoptosis of monocytes has not been reported so far. Systematic bioinformatics analysis indicated that *FOXO1* might be a target gene for miR-582-5p and its 3′UTR contains potential binding sites for miR-582-5p. To determine whether miR-582-5p could influence FOXO1 expression, miR-582-5p mimics or negative control of microRNA mimics were transfected into THP-1 cells. RT-PCR and western blot analysis showed that the miR-582-5p could suppress both FOXO1 mRNA and protein expression. Co-transfection of miR-582-5p and *FOXO1* 3′UTR-luciferase reporter vector into cells demonstrated that significant decrease in luciferase activity was only found in reporter vector that contained a wild type sequence of *FOXO1* 3′UTR, suggesting that miR-582-5p could directly target FOXO1. In conclusion, miR-582-5p inhibited apoptosis of monocytes by down-regulating FOXO1 expression and might play an important role in regulating anti-*M. tuberculosis* directed immune responses.

## Introduction

Tuberculosis (TB) is the second most common cause of death from an infectious disease after AIDS [1]. In 2011 alone, there are estimated 8.7 million new cases and 16.2 million existing cases of TB, and 1.4 million people died from TB [1]. *Mycobacterium tuberculosis* is the etiological agent of TB and it most often infects lungs but can also affect most parts of our body [2], [3].

During lung infection, *M. tuberculosis* first encounters host innate immune defense, such as alveolar macrophages [3], [4]. *M. tuberculosis* is able to circumvent the macrophage killing machinery by blocking the fusion of mycobacterial phagosome with lysosome and replicates within macrophages [5], [6]. It has been reported that virulent *M. tuberculosis* strain inhibits macrophage apoptosis and induces necrosis to spread the infection, while attenuated *M. tuberculosis* strain induces apoptosis, suggesting that macrophage apoptosis is a host innate defense mechanism against TB [7], [8], [9], [10], [11]. The hypothesis is supported by the observation that apoptotic vesicles from mycobacteria-infected macrophages stimulate CD8 T cells in vivo and limit *M. tuberculosis* replication [12].

Patients with active TB have increased frequency of peripheral blood monocytes compared with healthy controls, and effective anti-TB chemotherapy can reverse the change [13]. These observations suggested that alteration in peripheral monocytes is associated with TB infection, however, the underlying mechanisms remain to be elucidated.

microRNAs are endogenous regulatory RNA molecules that may regulate as much as 1/3 of encoding genes [14], [15]. They involve in a wide range of biological functions and act by binding to complementary sequences in the 3′ untranslated regions (UTR) of target genes to cause mRNA degradation or translational repression [14], [15]. To understand whether microRNAs play a role in regulating apoptosis of monocytes/macrophages in patients with TB, we investigated microRNAs that are differentially expressed between patients with active TB and healthy controls. Our study demonstrated that miR-582-5p was mainly expressed in human monocytes and it could inhibit apoptosis of monocytes by targeting FOXO1.

## Materials and Methods

### (1) Ethics statement

The study protocols were approved by the Ethics Committee of Beijing 309 Hospital (#20110311) and informed written consent was obtained from all participants.

### (2) Human subjects

One hundred and nine patients with active pulmonary TB (mean age 40.5±17.8, male/female: 61/48) were recruited from the TB Clinical Center of the Institute of Tuberculosis, 309 hospital, Beijing, China (Table 1). They were diagnosed as pulmonary TB based on results from acid fast staining of sputum smear/bacterial culture, chest X-ray examination, as well as clinical symptoms and responses to anti-TB chemotherapy. All TB patients were HIV-negative and had no immunocompromised conditions.

**Table 1:** Demographic and clinical characteristics of patients with active TB and controls.

	TB patients (n = 109)	Controls (n = 99)
Age (mean±SD)	40.5±17.8	38.97±11.26
Sex (male/female)	61/48	46/53
Pulmonary TB	109/109	No
New TB patients	60/109	N/A
MDR/XDR TB	6/109	N/A
Tuberculous pleurisy	11/109	No
Bronchial tuberculosis	28/109	No
Lung cavity (%)	65/109 (59.6%)	N/A
Fever (%)	48/109 (44%)	No
Elevated ESR^1^ (%)	60/109 (55%)	ND
Monocytes (absolute number)^2^	0.49±0.29×10^3^/µl	0.45±0.14×10^3^/µl
CD14^+^ monocytes^3^ (%)	7.92±2.13	5.5±1.48
BCG vaccinated	Yes	Yes
HIV positive	0	0

Note: ^1^ Erythrocyte sedimentation rate; ^2^The absolute number of monocytes was determined by white blood cell count; ^3^% of CD14^+^ Monocytes was determined by flow cytometry.

Ninety-nine healthy controls (mean age 38.97±11.26, male/female: 46/53) were recruited randomly from individuals underwent regular health check-up, with following inclusion criteria: (1) no fever, cough or other signs of active TB; (2) with normal physical examination result and normal radiography; (3) without HIV infection (Table 1).

### (3) Purification and culture of primary human monocytes

Blood samples for purification of peripheral blood mononuclear cells (PBMCs) were drawn at 7 to 8 am from patients with active TB and healthy controls. PBMCs were purified by density gradient centrifugation using Ficoll-Paque (GE Biosciences, Pittsburgh, PA, USA) within 6 hrs of blood collection. Anti-human CD3 and CD33 magnet beads (Miltenyi Biotec Inc., Auburn, CA, USA) were used to separate primary human monocytes according to manufacturer' s instructions [16].

Human monocytic cell line THP-1 (TIB-202) and human embryonic kidney 293T cells (CRL-11268) were obtained from the American Type Culture Collection (Manassas, VA, USA). Primary monocytes and THP-1 cells were cultured in RPMI 1640 medium (Invitrogen, Carlsbad, CA) supplemented with 10% fetal bovine serum (FBS), 100 U/ml penicillin, 100 µg/ml streptomycin and 2 mmol/L glutamine. HEK-293T cells were cultured in Dulbecco's Modified Eagle's Medium (Invitrogen, Carlsbad, CA) containing 10% fetal bovine serum (FBS), 100 U/ml penicillin, 100 µg/ml streptomycin and 2 mmol/L glutamine. All cells were incubated in 5% CO_2_ atmosphere at 37°C.

### (4) Surface antibody staining and flow cytometric analysis

For surface staining, PBMCs from TB patients and controls were stained with PE-CF594-labeled anti-human CD3 mAb (clone UCHT1, BD Biosciences, San Diego, California, USA), FITC--labeled anti-human CD14 mAb (clone HCD14, BioLegend, San Diego, CA, USA) or PE-Cy5-labeled anti-human CD33 mAb (clone WM53, BioLegend) for 30 min at 4°C. Appropriate isotype-matched control antibodies were used to determine background levels of staining. At least 100,000 events were collected and analyzed with Beckman CXP software on a FC-500 Flow Cytometer (Beckman Coulter, Brea, CA, USA).

### (5) RNA extraction and real-time RT-PCR

microRNAs were extracted by using a miRVana™ miRNA isolation kit (Applied Biosystems, Carlsbad, CA, USA) and cDNA synthesis were performed with TaqMan® microRNA reverse transcription kit (Applied Biosystems) according to manufacturer's protocols. TaqMan® microRNA assay for miR-582-5p (ID# 001983, Ambion, Carlsbad, CA, USA,) and TaqMan® universal PCR master mix (Applied Biosystems) were used for specific amplification and identification of miR-582-5p. Real-time RT-PCR was performed by using iQ5™ instrument (Bio-Rad, Hercules, CA, USA) with following conditions: 50°C for 2 min and 95°C for 10 min, followed by 40 cycles at 95°C for 10 s and 56°C for 30 s. The relative amount of microRNAs was normalized against U6 snRNA (ID#1973, Applied Biosysterms), and the fold change for the miR-582-5p was calculated by the 2-ΔΔCt method.

For detection of mRNA expression of *FOXO1*, the following primers were used: *FOXO1* forward (5′-GGATGGCATGTTCATTGAGCG-3′) and *FOXO1* reserve, (5′-ACTGCTTCTCTCAGTTCCTGC-3′). The expression levels were normalized by housekeeping gene *GAPDH* with following primer pair: *GAPDH* forward (5′-CCGCATCTTCTTTTGCGTCG-3′) and reserve (5′-TTCCCGTTCTCAGCCTTGAC-3′). Real-time RT-PCR was performed at following condition: 50°C for 2 min and 95°C for 10 min, followed by 40 cycles at 95°C for 10 s and 60°C for 30 s. The relative amounts of mRNA were calculated by the 2-ΔΔCt method.

### (6) Transfection of THP-1 cells

For transient transfection, 100 nmol/L of synthesized oligonucleotides, including miR-582-5p mimics and negative control of mimics (mimics NC) (both are from Ambion), *FOXO1* siRNA and negative control of siRNA (Ribobio, Guangzhou, China) were mixed with 100 µl Amaxa nucleofector solution (Lonza, Cologne, Germany) and transfected into 2×10^6^ cells by electroporation using Nucleofector II instrument (Lonza). A control plasmid pmaxGFP® (Lonza) which encodes EGFP was used to determine transfection efficacy. After transfection, the cells were allowed to recover for 6 hrs at 37°C and fresh RPMI 1640 medium was changed thereafter. The cells were cultured for additional 24 h in a 5% CO_2_ atmosphere at 37°C.

### (7) Cell apoptosis assay

Annexin V apoptosis detection kit I (BD Pharmingen™, San Diego, CA, USA) was used to determine apoptosis of the cells. In brief, cells transfected with miR-582-5p mimics or mimics NC were resuspended in 100 µl binding buffer, at a density of 1×10^6^ cells/ml, and were incubated with annexin V-FITC and PI for 15 min. The cells were analyzed with Beckman CXP software on a FC-500 Flow Cytometer (Beckman Coulter) within 1 hr of cell collection. At least 20,000 cells were counted in each assay.

For induced apoptosis assay of primary monocytes, PBMCs from patients with active TB and healthy controls were cultured in RPMI 1640 medium (Invitrogen) supplemented with 2% FBS, 100 U/ml penicillin, 100 µg/ml streptomycin and 2 mmol/L glutamine in a 5% CO_2_ atmosphere at 37°C for 24 hrs. The cells were collected and stained with PE/Cy7-labeled anti-human CD14 mAb (clone HCD14, BioLegend) and Annexin V apoptosis detection kit. Apoptotic rate of monocytes was determined by flow cytometric analysis.

### (8) Target genes prediction and gene ontology analysis

The target genes of miR-582-5p were predicted using microRNA analysis software TargetScan (http://www.targetscan.org/) and miRanda (http://www.microrna.org/). To investigate the biological processes that correlated with miR-582-5p expression, predicted target genes of miR-582-5p were projected to Gene Ontology analysis (http://david.abcc.ncifcrf.gov).

### (9) Vector construction and reporter assays

A DNA fragment of *FOXO1* 3′UTR region that contains potential target sequence of miR-582-5p was amplified by PCR using the following primer pair: ACTAGT ACCAGCTGTAAGTTGTGCATTG (forward primer) and AAGCTT TGCTGTGCACCTGTTCTCTT (reverse primer). The restriction sites *Spe*I and *Hin*dIII were included in the primers to facilitate cloning. The amplified products were inserted into pMIR-report vector (Ambion) and the resulting vector was designated pMIR-FOXO1-3′UTR-wt.

The *FOXO1* 3′UTR mutation oligos were synthesized as following: 5′- CTAGTTTTCTTTAGCCTGTAGCAACCTACCCTAAAATTCCTATCATTATGTA-3′ (sense) and 5′ AGCTTACATAATGATAGGAATTTTAGGGTAGGTTGCTACAGGCTAAAGAAAA-3′ (antisense). The mutated bases in the potential target sequence of miR-582-5p were indicated in the box, and restriction sites *Spe*I and *Hin*dIII were included in the oligos. The complementary oligos were annealed and inserted into pMIR-report vector, and the resulting vector was designated pMIR-FOXO1-3′UTR-mut.

For luciferase assay, HEK-293T cells were seeded onto 96-well plates (1×10^4^ cells per well) 24 h before transfection. 100 ng pMIR-FOXO1-3′UTR-wt or pMIR-FOXO1-3′UTR-mut was co-transfected with 50 nmol/L miR-582-5p mimics or mimics NC (Ambion) into 293T cells by using Lipofectamine 2000 (Invitrogen). Cells lysates were prepared with Passive Lysis Buffer (Promega, Madison, WI, USA) 24 h after transfection, and luciferase activities were measured by using the Dual Luciferase Reported Assay Kit (Promega) on a luminometer.

### (10) Western blot analysis

Protein expression levels of FOXO1 in THP-1 cells transfected with miR-582-5p mimics or mimics NC were determined by western blot. Briefly, THP-1 cells were lysed with RIPA buffer (Beyotine, Jiangsu, China) and the protein concentration was determined using the BCA protein assay kit (Vigorous, Beijing, China). Twenty microgram total protein were electrophoresed on a 10% (w/v) SDS-PAGE gel (Bio-Rad) and transferred onto a PVDF membrane (Millipore, Brussels, Belgium). After blocking with 5% (w/v) BSA, membranes were probed with the primary antibody against FOXO1 (Santa Cruz Biotechnology, Dallas, Texas, USA) and then incubated with horseradish peroxidase-conjugated secondary antibody (Santa Cruz Biotechnology). The membranes were developed with western blotting luminol reagent (Santa Cruz Biotechnology). Data were normalized to the housekeeping protein β-actin (Santa Cruz Biotechnology).

### (11) Statistical analysis

All statistical analyses were performed using the Graphpad Prism 5.0 software package. Data were shown as mean±SD or median (25% percentile, 75% percentile). For comparison between two groups, a two-tailed unpaired t-test or Mann-Whitney test were used. Differences were considered significant at a level of *p*<0.05.

## Results

### (1) Patients with active TB had elevated frequency of monocytes in peripheral blood

**Figure 1:**
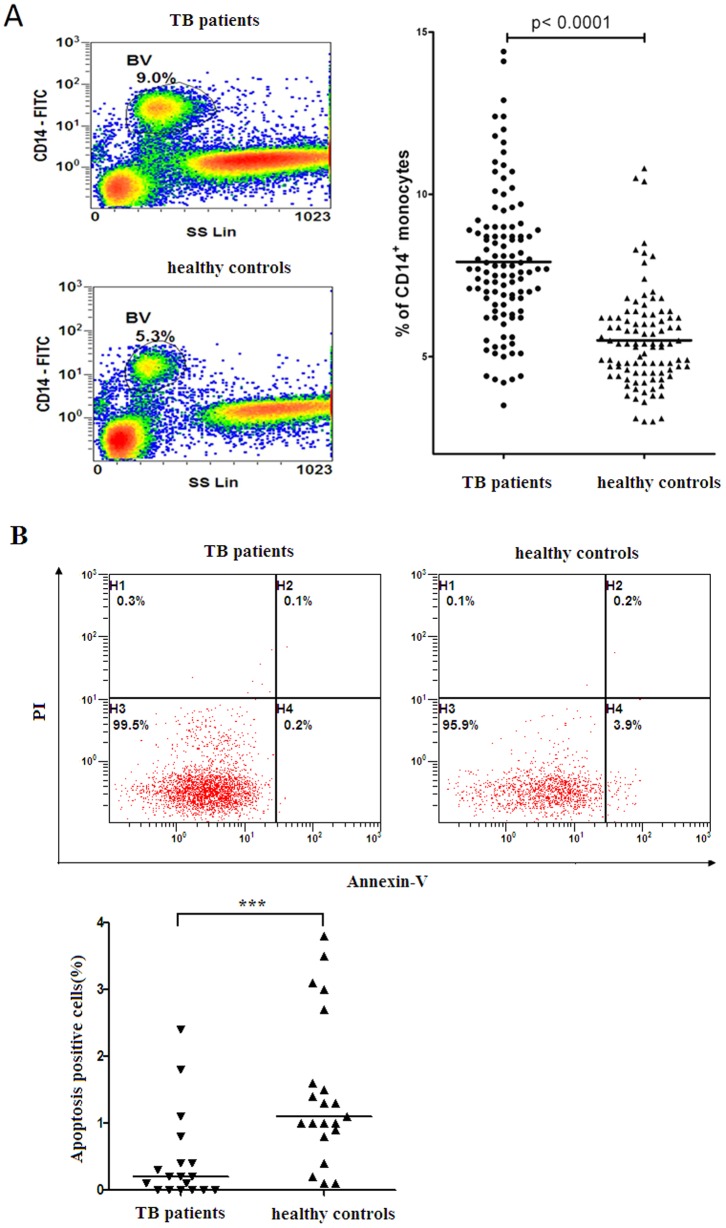
Frequencies and apoptosis of CD14^+^ peripheral blood monocytes in patients with active TB and healthy controls. (A) Representative flow cytometric plots showing gating strategy and percentage of monocytes (left panel). Monocytes were defined by high CD14 expression (gate BV). Patients with active TB had significantly elevated frequency of CD14^+^ monocytes compared with healthy controls. Two-tailed unpaired t-test was used for statistical analysis between groups. (B) Representative flow cytometric plots showing apoptosis of monocytes (upper panel). PBMCs from patients with active TB and healthy controls were incubated with RPMI-1640 contained 2% FBS for 24 h at 37°C, and cells were stained with fluorochrome-labeled anti-human CD14, Annexin V and PI. Cells that were positive for Annexin V were defined as apoptosis. Monocytes from patients with active TB had significantly lower percentage of apoptotic cells than that from healthy controls. Mann-Whitney test was used for statistical analysis between groups.

Peripheral blood was obtained from 109 patients with active TB and 99 healthy controls, and frequencies of monocytes were determined by flow cytometric analysis based on CD14 expression (Fig. 1A). Patients with active TB had significantly elevated frequency of CD14^+^ monocytes compared with healthy controls (Fig. 1A) (p<0.0001), and the result was consistent with previous reports [13].

### (2) Monocytes from patients with active TB had decreased apoptosis

To determine if there was any alteration in apoptosis of monocytes in patients with active TB, fresh circulating monocytes from both patients with active TB and healthy controls were cultured in RPMI 1640 contained 2% FBS for 24 hrs. The cells were stained with fluorochrome-labeled anti-human CD14, Annexin-V and PI, and the apoptotic rate of monocytes was analyzed by flow cytometry (Fig. 1B). The percentage of apoptotic monocytes was 0.2% (0.0%, 0.5%) in patients with active TB and 1.1% (0.85%, 2.15%) in healthy controls (Fig. 1B). Statistical analysis indicated that the difference in apoptosis of monocytes between patients with active TB and healthy controls was highly significant (p<0.001) (Fig. 1B). The results suggested that circulating monocytes from patients with active TB were more tolerant to apoptosis than that from healthy controls.

### (3) miR-582-5p was mainly expressed in monocytes and was upregulated in patients with active TB

To understand whether microRNA can modulate apoptosis of monocytes, we investigated differentially expressed microRNAs in patients with active TB. Our previous microRNA array analysis showed that miR-582-5p was significantly upregulated in patients with active TB compared with healthy controls [16].

**Figure 2:**
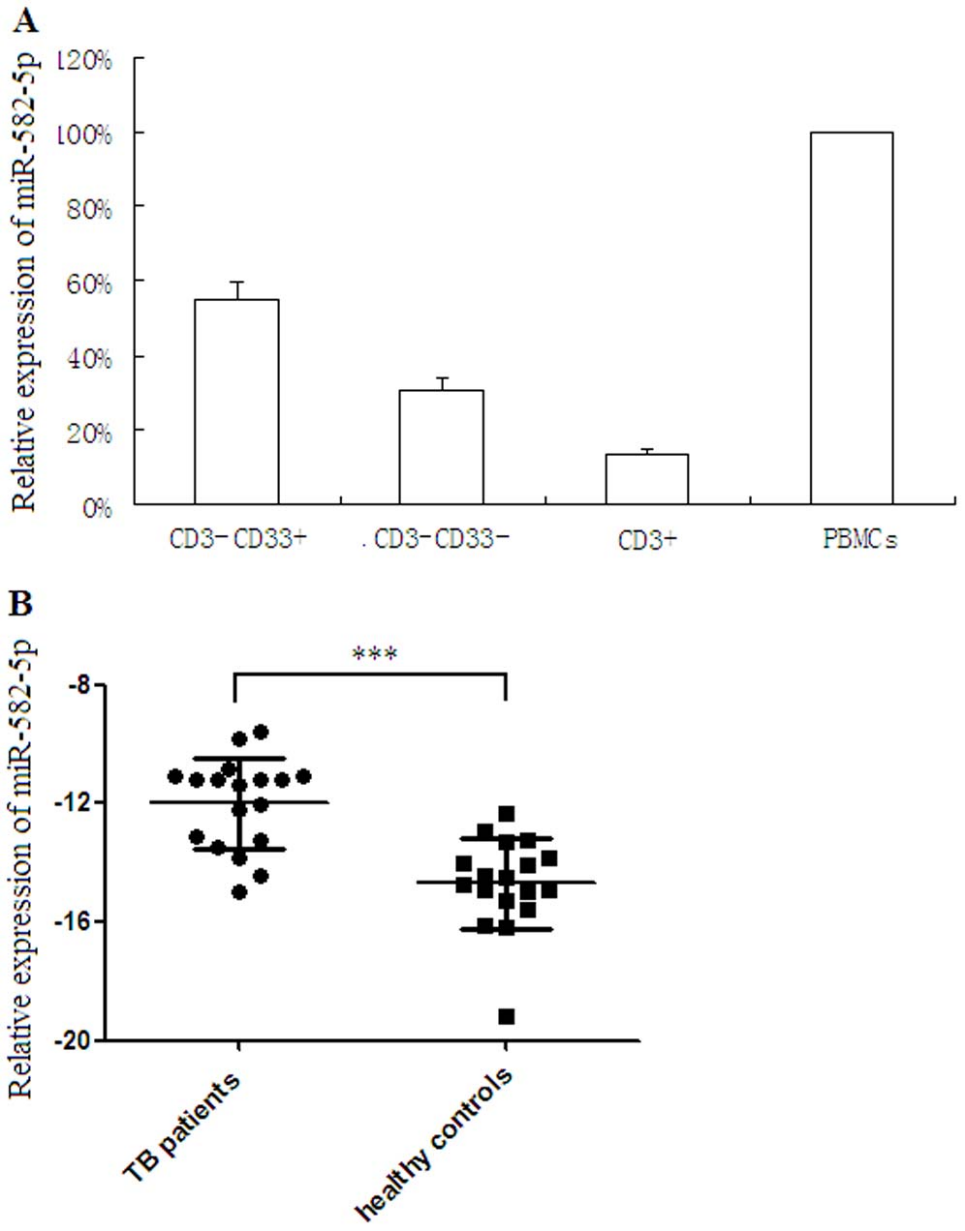
miR-582-5p expression detected by real-time RT-PCR. (A) Relative expression of miR-582-5p in CD3^−^CD33^+^, CD3^−^CD33^−^, CD3^+^ cell subsets and in PBMCs from patients with active TB (n = 3). miR-582-5p was mainly expressed in CD3^−^CD33^+^ cells. (B) Patients with active TB had significantly higher expression of miR-582-5p as compared with healthy controls. The relative expression of miR-582-5p, as defined by mean -ΔCt, was normalized against U6 snRNA. Two-tailed unpaired t-test was used for statistical analysis between groups.

To determine the expression pattern of miR-582-5p in human blood cells, fresh PBMCs were separated by anti-human CD3 magnetic beads into CD3^+^ and CD3^−^ cell population, and the CD3^−^ cells were further separated by anti-human CD33 magnetic beads into CD33^+^ and CD33^−^ subsets. The relative expression of miR-582-5p in different cell subsets was examined by real-time RT-PCR with U6 snRNA as an internal control to normalize the relative amount of miR-582-5p expression. CD3^−^CD33^+^ cell subset was found to be the main cell type that expressed miR-582-5p (Fig. 2A). The result indicated that miR-582-5p was mainly expressed in monocytes.

The expression level of miR-582-5p was compared between 18 patients with active TB and 18 healthy controls. As shown in figure 2B, mean miR-582-5p expression level (−ΔCt) was −12.005±1.527 for patients with active TB and −14.698±1.537 for healthy controls (p<0.0001). The result indicated patients with active TB had significantly higher expression of miR-582-5p as compared with healthy controls.

### (4) miR-582-5p inhibited apoptosis of monocytes

To explore the possible function of miR-582-5p, its target genes were predicted by two computational algorithms (TargetScan and miRanda) which predicted that 1157 genes could be targeted by miR-582-5p, and some of the genes are related to cell proliferation and apoptosis (Table 2).

**Table 2:** List of predicted target genes of miR-582-5p.

Functional class	Number	Representative target genes (mirSVR score)
Cell proliferation and apoptosis	77	UBR5(−1.3061), RUNX3(−1.2152), CD81(−0.9453), FOXO1(−0.9050), MNT(−0.6641), NAP1L1(−0.5536), MXD1(−0.4865), NOTCH1(−0.4284), CDKN2B(−0.3181), BTG2(−0.2992), MYCN(−0.2823), KRAS(−0.2733), CDKN1C(−0.1806), HHIP(−0.1069).
Cell cycle	72	STRN3(−1.2464), STARD13(−1.1878), SMC(−1.1666), PARD6B(−1.1054), BOLL(−1.0666), CHFR(−1.0134), DBC1(−0.8010), CKAP2(−0.6645), PTPN11(−0.5345), CCPG1(−0.5116), VEGFC(−0.2079), DSN1(−0.1560), ACVR1B2(−0.1378), EGFL6(−0.1178).
RNA metabolic process	257	ZMYM2(−1.3374), CHD7(−1.2727), CREM(−1.2723), HIVEP1(−1.2150), MDS1(−1.2017), SMC3(−1.1666), DICER1(−0.6541), MED14(−0.5179), MED2(−0.4285), PARP15(−0.3190), HARS2(−0.2713), FOXP2(−0.1968), MNDA(−0.154), MGA(−0.1506).
Transcription	230	BL2F1(−1.2488), GTF2B(−1.108), PIAS2(−0.9649), VSX2(−0.9341), PGBD1(−0.7494), TLX2(−0.5704), HMGB3(−0.4069), ZBTB10(−0.3016), TFAP4(−0.1921), HNF4G(−0.1931), CDYL(−0.188), PRDM2(−0.1876), TBL1X(−0.1551), PBX3(−0.1113).
Developmental process	259	TCF12(−1.1329), FGFR2(−1.094), SHROOM2(−0.7891), NDUFS1(−0.7382), CHOOL(−0.6836), UBE2B(−0.5968), MAP3K5(−0.5892), MLL2(−0.4141), MAP1B(−0.3801), ABTB2(−0.2395), DYRK2(−0.1878), COL11A1(−0.1607), GJA1(−0.1537), MCOLN3(−0.1425).
Signal transduction	255	NFE2L2(−1.2811), MARK1(−1.1437), GRIA4(−1.133), CXCL2(−1.12), FGR(−1.0019), TNFRSF21(−0.8999), MED14(−0.3114), CD3D(−0.246), CDKN1C(−0.1806), TULP4(−0.1334), TNFRSF17(−0.1014).
Cell differentiation	145	BOLL(−1.066), ADRB1(−0.9266), PTPRJ(−0.8375), PAX8(−0.7105), PRKAA1(−0.6727), CKAP2(−0.6645), EIF2AK3(−0.5779), SOX2(−0.2609), STK3(−0.1377).
Neurogenesis	37	ROBO2(−1.2281), SMARCA4(−1.1566), NRP1(−1.0410), MDGA2(−0.9240), GNAO1(−0.7466), NRTN(−0.3797), RUNX1(−0.1997), CDKN1C(−0.1806), DLX5(−0.1455), EFNB3(−0.1288).
MAPKKK cascade	20	MAP3K7(−1.1261), MAP4K3(−0.9563), GHRL(−0.7419), BIRC7(−0.4401), MAP3K5(−0.3776), MAPK14(--0.365), CAV3(−0.234), NF1(−0.1859), MAPK1(−0.1768), TGFB3(−0.111).
Cell migration	33	CD24(−1.1713), PAFAH1B1(−1.1234),MDGA2(−0.9240), MAP3K1(−0.8100), PARD6B(−0.7454), LMXIA(−0.5839), ABHD2(−0.4794), APP(−0.3646), NR2F2(−0.3644).
Protein modification process	131	UBE2E1(−1.17), FGFR2(−1.0554), USP24(−0.8081), UBR1(−0.7731), PRKC2(−0.7384), PTPN9(−0.4113), UGGT2(−0.2751), IKBKAP(−0.2014), CSNK1E(−0.1131), PARP8(−0.1083).
Response to stress	78	EPC2(−1.3171), SFPQ(−1.024), ADRB1(−0.9266), SNN(−0.4698), CDKN2B(−0.3181), PENK(−0.1887), ADAM17(−0.1651), ESCO1(−0.1605), HSPA14(−0.1468).

**Figure 3:**
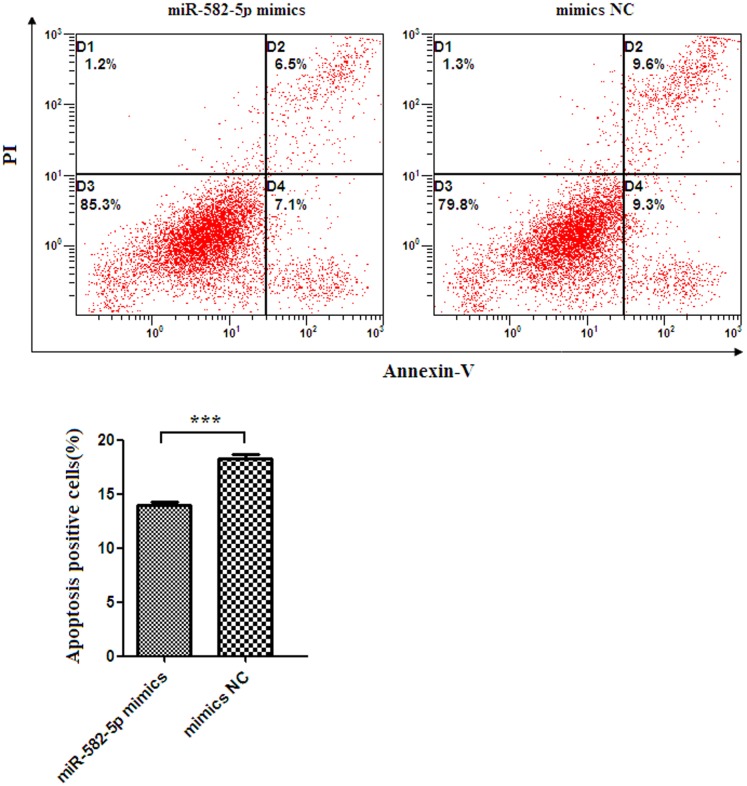
Inhibition of apoptosis in monocytes by miR-582-5p. (A) Representative flow cytometric plots showing apoptotic percentage of THP-1 cells transfected with miR-582-5p mimics or negative control of mimics (mimics NC). (B) The apoptotic percentage of THP-1 cells transfected with miR-582-5p mimics was significantly lower than those transfected with negative control of microRNA mimics (mimics NC).

To investigate whether miR-582-5p can influence apoptosis of monocytes, miR-582-5p mimics or negative control of microRNA mimics were transfected into human monocytic cell line THP-1 cells. The reason to use THP-1 cell line instead of primary monocytes was due to low transfection efficiency of fresh human monocytes. As shown in figure 3, the apoptotic rate of THP-1 cells transfected with miR-582-5p mimics was 13.9%±0.5%, which was significantly lower than those transfected with negative control of microRNA mimics (18.3%±0.7%) (Fig. 3) (*p*<0.001). These data suggested that miR-582-5p could inhibit apoptosis of monocytes.

### (5) miR-582-5p targeted FOXO1 to inhibit apoptosis

**Figure 4:**
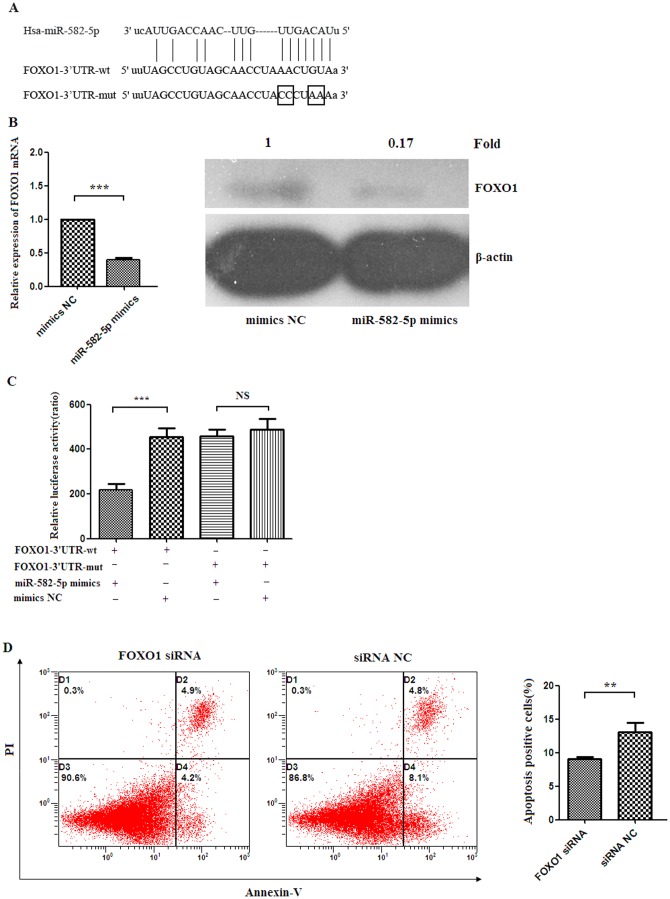
*FOXO1* is a direct target of miR-582-5p. (A) Alignment between the predicted miR-582-5p target site of *FOXO1* 3′UTR region and miR-582-5p. (B) Real time RT-PCR and western blot analysis showing *FOXO1* mRNA and protein expression levels in THP-1 cells transfected with miR-582-5p mimics or mimics NC, respectively. (C) Co-transfection of miR-582-5p mimics/mimics NC and *FOXO1* 3′UTR-luciferase reporter vector into HEK-293T cells demonstrated that significant decrease in luciferase activity was only found in reporter vector that contained a wild type sequence (FOXO1-3′UTR-wt), not in vector that contained a mutant sequence (FOXO1-3′UTR-mut) within the miR-582-5p binding site. Values were presented as relative luciferase activity after normalization to Renilla luciferase activity. FOXO1-3′UTR-wt: pMIR-FOXO1-3′UTR-wt vector; FOXO1-3′UTR-mut: pMIR-FOXO1-3′UTR-mut vector. (D) Representative flow cytometric plots showing apoptotic ratio of THP-1 transfected with *FOXO1* siRNA or negative controls of siRNA (siRNA NC) (left panel). The apoptotic percentage of THP-1 cells transfected with *FOXO1* siRNA were significantly lower than those transfected with negative control siRNA (siRNA NC) (*p*<0.01).

Systematic bioinformatics analysis indicated that *FOXO1* might be a target gene of miR-582-5p and its 3′UTR contains potential binding sites for miR-582-5p (Fig. 4A). To determine whether miR-582-5p can influence FOXO1 expression, miR-582-5p mimics or negative control of microRNA mimics were transfected into THP-1 cells. Real time RT-PCR and western blot analysis showed that miR-582-5p could suppress both *FOXO1* mRNA and protein expression (Fig. 4B).

To further determine whether 3′UTR of *FOXO1* contains binding sites for miR-582-5p, the 3′UTR of *FOXO1* gene was cloned into a luciferase reporter vector, pMIR-report. As shown in Figure 4C, co-transfection of miR-582-5p mimics/mimics NC and *FOXO1* 3′UTR-luciferase reporter vector into cells demonstrated that significant decrease in luciferase activity was only found in reporter vector pMIR-FOXO1-3′UTR-wt that contained a wild type sequence, not in vector pMIR-FOXO1-3′UTR-mut that contained mutations within the miR-582-5p binding site (Fig. 4C). These data indicated that 3′UTR of *FOXO1* is a target gene of miR-582-5p and contains binding sites for miR-582-5p.

Next, siRNA-mediated FOXO1 silencing was performed to investigate the influence of FOXO1 on apoptosis of monocytes. As shown in Figure 4D, the apoptotic rate of THP-1 cells transfected with *FOXO1* siRNA (9.1%±0.3%) were significantly lower than those transfected with negative control siRNA (13.1%±1.4%) (*p*<0.01).

Taken together, these results indicated that miR-582-5p could inhibit apoptosis of monocytes by directly down-regulating FOXO1.

## Discussion

Interactions between *M. tuberculosis* and host determine the outcome of TB [4], [6], [17] and macrophage apoptosis is a host innate defense mechanism against TB [7], [8], [9], [10], [11]. In this study, we found that peripheral blood monocytes from patients with active TB had significantly lower percentage of apoptotic cells than that from healthy controls. The result indicates that *M. tuberculosis* infection can modulate apoptosis of monocytes, which might play roles in host immunity against TB and augmented circulating monocytes in TB patients [13].

Many studies have shown that microRNAs play a crucial role in regulation cellular proliferation and apoptosis, and they might have great potential in cancer treatment [18], [19], [20]. To determine if microRNAs can regulates apoptosis of monocytes/macrophages in TB patients, we performed a systematic analysis of microRNAs that are differentially expressed between patients with active TB and healthy controls. miR-582-5p was one of microRNAs that was selected for further analysis based on target gene prediction. We demonstrated that miR-582-5p was upregulated in patients with active TB and it could inhibit apoptosis of monocytes. To our knowledge, the role of miR-582-5p in regulating apoptosis of monocytes has not been reported so far.

FOXO1 is a transcription factor of Forkhead box O (FoxO) family that involves in diverse functions of cellular processes [21], [22], [23], [24], [25], [26]. Previous studies found that FOXO1 promotes apoptosis in many cell types and is suggested as a tumor suppressor [22], [24], [27], [28], [29]. It has been shown that FOXO1 is highly expressed in normal germinal center B cells and is not expressed in classical Hodgkin lymphoma (cHL), and ectopic expression of a constitutively active FOXO1 induces apoptosis in cHL cell lines and blocks proliferation [25]. The function of FOXO1 is regulated by multiple mechanisms, such as AKT/PKB and MAPK/ERK kinases, Sirtuin 1 (SIRT1), CDK2 and up-regulation of microRNA miR-370 [22], [23], [26].

Through systematic bioinformatics analysis, we found that 3′UTR of *FOXO1* contains potential binding sites for miR-582-5p and might be one of its target genes. The relationship between miR-582-5p and *FOXO1* has not been reported before. Our study demonstrated that miR-582-5p can directly target 3′UTR of *FOXO1* to inhibit apoptosis of monocytes. The observation indicates that miR-582-5p inhibits apoptosis of monocytes by targeting FOXO1.

In summary, miR-582-5p was upregulated in monocytes from patients with active TB and could inhibit apoptosis of monocytes. *FOXO1* was a target gene of miR-582-5p, which participated in regulation of monocyte apoptosis.
